# Activated Allogeneic NK Cells Preferentially Kill Poor Prognosis B-Cell Chronic Lymphocytic Leukemia Cells

**DOI:** 10.3389/fimmu.2016.00454

**Published:** 2016-10-27

**Authors:** Diego Sánchez-Martínez, Pilar M. Lanuza, Natalia Gómez, Aura Muntasell, Elisa Cisneros, Manuela Moraru, Gemma Azaceta, Alberto Anel, Luis Martínez-Lostao, Martin Villalba, Luis Palomera, Carlos Vilches, José A. García Marco, Julián Pardo

**Affiliations:** ^1^Biomedical Research Center of Aragón (CIBA), Aragón Health Research Institute (IIS Aragón), University of Zaragoza, Zaragoza, Spain; ^2^Immunogenetics and HLA, Instituto de Investigación Sanitaria Puerta de Hierro, Majadahonda, Spain; ^3^Immunity and infection Lab, IMIM (Hospital del Mar Medical Research Institute), Barcelona, Spain; ^4^Hospital Clínico Universitario Lozano Blesa, Instituto Aragonés de Ciencias de la Salud (IACS)/Aragón Health Research Institute (IIS Aragón), Zaragoza, Spain; ^5^Department of Biochemistry and Molecular and Cellular Biology, Aragón Health Research Institute (IIS Aragón), University of Zaragoza, Zaragoza, Spain; ^6^Nanoscience Institute of Aragon (INA), University of Zaragoza, Zaragoza, Spain; ^7^INSERM U1183, Université de Montpellier 1, UFR Médecine, Montpellier, France; ^8^Institute for Regenerative Medicine and Biotherapy (IRMB), CHU Montpellier, Montpellier, France; ^9^Unidad de Citogenética Molecular/Servicio de Hematología, Hospital Universitario Puerta de Hierro-Majadahonda, Madrid, Spain; ^10^Aragón I+D Foundation (ARAID), Government of Aragon, Zaragoza, Spain; ^11^Department of Microbiology, Preventive Medicine and Public Health, University of Zaragoza, Zaragoza, Spain

**Keywords:** allogeneic NK cells, bad prognosis leukemia, mismatch, chronic lymphocytic leukemia, leukemia resistance

## Abstract

Mutational status of *TP53* together with expression of wild-type (wt) *IGHV* represents the most widely accepted biomarkers, establishing a very poor prognosis in B-cell chronic lymphocytic leukemia (B-CLL) patients. Adoptive cell therapy using allogeneic HLA-mismatched Natural killer (NK) cells has emerged as an effective and safe alternative in the treatment of acute myeloid and lymphoid leukemias that do not respond to traditional therapies. We have described that allogeneic activated NK cells eliminate hematological cancer cell lines with multidrug resistance acquired by mutations in the apoptotic machinery. This effect depends on the activation protocol, being B-lymphoblastoid cell lines (LCLs) the most effective stimulus to activate NK cells. Here, we have further analyzed the molecular determinants involved in allogeneic NK cell recognition and elimination of B-CLL cells, including the expression of ligands of the main NK cell-activating receptors (NKG2D and NCRs) and HLA mismatch. We present preliminary data suggesting that B-CLL susceptibility significantly correlates with HLA mismatch between NK cell donor and B-CLL patient. Moreover, we show that the sensitivity of B-CLL cells to NK cells depends on the prognosis based on *TP53* and *IGHV* mutational status. Cells from patients with worse prognosis (mutated *TP53* and wt *IGHV*) are the most susceptible to activated NK cells. Hence, B-CLL prognosis may predict the efficacy of allogenic activated NK cells, and, thus, NK cell transfer represents a good alternative to treat poor prognosis B-CLL patients who present a very short life expectancy due to lack of effective treatments.

## Introduction

B-cell chronic lymphocytic leukemia (B-CLL), a heterogeneous disease with variable clinic presentation and evolution, is the most common leukemia in adults in Western World (1, 2). It is characterized by the accumulation of CD5^+^ B cells in peripheral blood and lymphoid organs (3). This leukemia is usually treated with chemotherapy and anti-CD20 antibodies (i.e., Rituximab) (4), but remains incurable largely due to development of refractory disease. This is frequently associated with the expression of molecular markers that confer bad prognosis (2).

Several prognostic and predictive markers have been described for B-CLL, including cytogenetic abnormalities like 17p and 11q deletions, associated in some instances with rapid clinical progression, chemotherapy resistance, and inferior survival (5). Expression of membrane-proteins such as CD38 (6) and CD49d (7) or intracellular ZAP70 (8) have also been described as adverse prognostic factors. Among them, a recent study indicates that CD49d expression is the best immunophenotypic predictor of the overall patient survival (9). Some single mutations like NOTCH1 (10, 11), the splicing factor 3b (SF3B1) (12, 13), BIRC3 (14), exportin 1 (*XPO1*), MYD88, and KLHL6 (15) have also been related to poor prognosis, although their utility as prognostic markers is still under clinical evaluation. Among all of them, *TP53* mutation/deletion and expression of unmutated *IGHV* are widely accepted as indicators of poor prognosis at the time of diagnosis (16–19).

Unmutated *IGHV* is associated with higher aggressiveness of B-CLL cells since proliferating signals through B cell receptor are unaffected. In contrast, mutated IGHV produces unresponsive B cell receptors. *TP53* is a tumor suppressor that plays a key role in DNA repair as well as apoptosis trigger in response to DNA damage. Thus, inactivation of *TP53* favors malignant cell transformation and confers resistance to chemo and radiotherapy (20).

Natural killer (NK) cells belong to the innate immune system and were originally identified as lymphocytes capable of killing cells that have downregulated MHC-I expression due to pathogen infection or transformation (21–26). They constitute a heterogeneous cell population with distinct phenotypic and functional characteristics, including, but not limited to, their ability to mediate cytolytic activity (27, 28). NK cell activity is regulated by the equilibrium between signals transduced by inhibitory and activating receptors, which dictates target cell elimination and pro-inflammatory cytokine production (29, 30). The main inhibitory receptors, NKG2A killer-cell immunoglobulin-like receptors (KIRs) family, bind to MHC-I molecules on target cells. The main activating receptors, NKG2D and NCRs (NKp30, NKp44, and NKp46) recognize stress ligands on target cells (31, 32). The balance between inhibitory and activating signals dictates if NK cells will recognize and destroy target cells.

During allogeneic hematopoietic stem cell transplantation, in a context of KIR–MHC mismatch, HLA alleles expressed on target cells may not inhibit NK cells. Accordingly, allogeneic NK cells have been proposed to kill hematological cancer cells and improve prognosis, mainly in the context of mismatched hematopoietic stem cell transplantation (33–37). Clinical protocols based on these concepts have been designed to treat some hematological malignancies, including lymphoma, acute myeloid and lymphoid leukemia, and multiple myeloma (34, 37–42). Regarding B-CLL, at present, it is unclear whether KIR–HLA mismatch may also regulate B-CLL allogeneic NK cell recognition. NK cells activated with high concentrations of IL-2, known as lymphokine-activated killer (LAK) cells, were shown to kill B-CLL cells (43–45). In contrast, other authors reported that autologous and allogeneic LAK cells were unable to kill B-CLL cells (46–48). More recently, it was shown that unstimulated NK cells did not kill B-CLL cells, but cytotoxicity was recovered using IL-15-activated NK cells in combination with rituximab (49). Clinical trials based on autologous NK cells have not shown benefits (50).

We have previously shown that the selection of a proper activating stimulus is critical to generate activated NK cells able to kill chemoresistant hematological cancer cell lines as well as cells from B-CLL patients (51, 52). Allogeneic NK cells activated in the presence of EBV-transformed B-cell lymphoblastoid cell lines (LCL) presented significantly higher cytotoxicity than those generated with K562 cells and IL-2/IL-15. This activation protocol has been now employed to (i) analyze the molecular determinants that drive allogeneic NK cell recognition of B-CLL cells and (ii) to test the susceptibility of adverse prognosis B-CLL cells, defined according to *IGHV* mutational status and *TP53* deletion/mutation, to allogeneic activated NK cells.

## Materials and Methods

### Isolation and Activation of Human NK Cells

Human *ex vivo* NK cells were enriched by using anti-CD56 MicroBeads with a MultiStand MACS (MACS, Miltenyi Biotec) from freshly isolated peripheral blood mononuclear cells (PBMCs).

Activation of human primary NK cells was pursued by culturing PBMCs for 5 days with Mitomycin C-treated R69- or 721.221-LCL at 10:1 PBMC:stimulator ratio. Subsequently, NK cells were enriched using anti-CD56 MicroBeads with a MultiStand MACS (MACS, Miltenyi Biotec).

PBMCs were obtained from blood from healthy donors by Ficoll gradient centrifugation (Blood and Tissue Bank of Aragon; approved by the CEICA, number: C.I.PI11/006). NK cell purity (CD56^+^/CD3^−^) was higher than 90% in all cases. Contamination with CD8^+^ CD3^+^ cells was less than 2%.

### B-CLL Patients

Blood samples from patients with B-CLL were obtained from Hospital Clinico Lozano Blesa (Zaragoza) and Hospital Puerta de Hierro-Majadahonda (Madrid). They were processed by Ficoll gradient centrifugation to obtain PBMCs and stored frozen in liquid nitrogen until their use. In all cases, the percentage of CD19^+^CD5^+^ cells was higher than 80% and no differences were observed between frozen and fresh B-CLL cells regarding their susceptibility to NK cells. This study was approved by Ethics Committee for Clinical Research of Aragon (CEICA), number: C.I.PI11/006; and by Ethics Committee for Clinical Research of Hospital Puerta de Hierro-Majadahonda, number: PI31_13.

### Analyses of *TP53* and *IGHV* Mutational Status

*IGHV* gene mutational status was analyzed and classified according to ERIC recommendations (53). *TP53* genetic abnormalities were detected by conventional cytogenetics, FISH, and Sanger sequencing analysis.

### NK Cell-Mediated Cytotoxicity

NK cells were labeled with 1 μM of CellTracker™ Green CMFDA (Life Technologies) and incubated with target cells at 9:1 e:t ratio for 4 h. Subsequently, phosphatidylserine (PS) translocation and membrane damage were analyzed in the green or violet fluorescence negative target cell population by flow cytometry using annexin V and 7AAD as previously described (51).

### Analyses of HLA Genotype and Prediction of KIR–HLA Mismatch

Killer-cell immunoglobulin-like receptor ligands were deduced from the cell HLA types, determined by standard methods approved by the European Federation for Immunogenetics, as in Ref. (54) or directly assessed by PCR with sequence-specific primers targeted to the critical polymorphic positions (oligonucleotide sequences and PCR conditions available upon request). Mismatch was defined, according to the missing-self model, as the absence in the target cell of a KIR ligand present in the effector cells.

### Flow Cytometry

The antibodies against HLA-E-PE (clone 3D12), HLA-ABC/E/F/G-FITC (clone W6/32), DR4-PE (clone DJR1), and DR5-PE (clone MD5-1) were from eBioscience. The antibody against ICAM-1-APC (clone HA58) was from BD. The antiCD19-APC (clone LT19) and antiCD5-FITC (clone UCHT2) were from Miltenyi Biotec. The NKR-Fc quimeras were from R&D Systems (NKG2D-Fc) or were provided by Miguel López-Botet (NKp30-Fc and NKp46-Fc). The anti-human IgG secondary antibody labeled with PE was from Jackson Immunoresearch. All antibodies were diluted in PBS with 5% FCS and 0.1% sodium azide for cell staining. To stain NKR ligands using NKR-Fc quimeras, cells were blocked with rabbit serum before staining with the respective chimeras.

### Statistical Analyses

Statistical analyses were performed using the GraphPad Prism v 4.0 software by one-way analysis of variance (ANOVA) as indicated in the figure/table legends. Regression-tree analyses (Decision tree learning) were used to classify B-CLL samples according to its susceptibility to NK cells. This is a predictive model that maps the values of cell death (percentage) to conclusions about categorical predictor variables.

## Results

### NK Cells Require Activation to Kill CLL Cells

**Figure 1:**
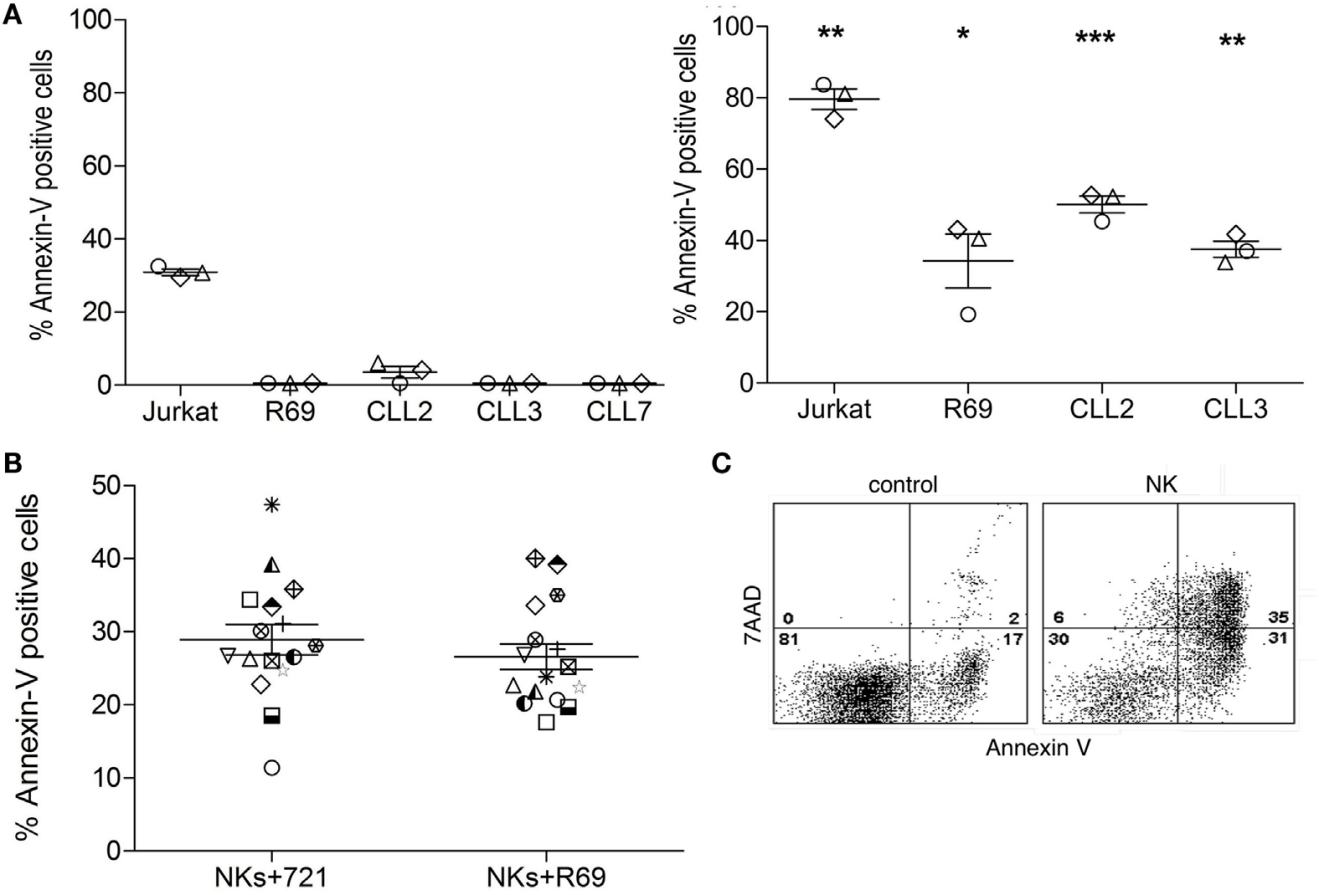
**Allogeneic NK cells require activation to kill B-CLL cells**. **(A)** Jurkat and R69 cells or cells from B-CLL patients were incubated with naïve (left) or R69-LCL activated (right) NK cells (MACS enriched, >90% CD56^+^CD3^−^ cells; CTGreen labeled) for 4 h at 9:1 effector:target ratio as described in Section “Materials and Methods.” Subsequently, PS traslocation (annexin V DY634) and membrane permeabilization (7AAD uptake) were analyzed by flow cytometry in the cell population negative for CTGreen. **(B)** Cells from B-CLL were incubated with R69- or 721-LCL-activated NK cells (MACS enriched, >90% CD56^+^CD3^−^ cells; CTGreen labeled) for 4 h at 9:1 effector:target ratio as described in Section “Materials and Methods.” Subsequently, PS traslocation (annexin V DY634) and membrane permeabilization (7AAD uptake) were analyzed by flow cytometry in the cell population negative for CTGreen. Data in the graphics are represented as the mean ± SEM from three independent NK cell donors **(A)** or from 14 independent B-CLL patients **(B)**. Annexin V^+^ cells represent the % of AnnexinV^+^7AAD^−^ plus AnnexinV^+^7AAD^+^ cells. Statistical analysis was performed by comparing the means of naive versus activated NK cells within each group using 2-way ANOVA with Tukey HSD *post hoc* test; ns, not significant, *p < 0.05, **p < 0.01, ***p < 0.001. **(C)** Representative dot plot of Annexin V/7AAD staining in a B-CLL sample incubated in medium alone (control) or together with NK cells (NK). Numbers correspond to the % of cells in each quadrant.

As mentioned above, it is unclear whether NK cells activated with exogenous cytokines may kill B-CLL cells. Our group has previously described that NK cells activated with EBV-transformed R69 or 721.211 LCLs are able to eliminate cells from B-CLL patients without requiring supplementation with exogenous cytokines (51). Importantly, activated NK cells did not kill healthy non-transformed PBMCs. Here, we confirmed that the cytotoxic potential of NK cells activated with R69 LCLs was increased in comparison with freshly isolated NK cells from the same donors. As shown in Figure 1A, freshly isolated allogeneic NK cells from healthy donors did not kill B-CLL cells. In contrast, activated NK cells presented a significantly increased cytotoxic potential against B-CLL cells, even in cells from a patient (CLL2) with chemotherapy and Rituximab resistance (Table 1). Killing of Jurkat cells and R69 LCL (that expresses all known HLA ligands for inhibitory KIRs) was also significantly enhanced after NK cell activation. Cell death was almost completely inhibited by EGTA indicating the granule exocytosis was the main mechanism employed by NK cells to kill B-CLL cells (data not shown). As previously found in NK cells activated with R69 LCLs (51), the NK cell receptors NKG2A, NKp30, NKp44, and DNAM and the cytotoxic protease granzyme B were upregulated in activated NK cells (data not shown).

**Table 1:** **Characteristics of CLL patients used in the study of compatibility**.

Sample	Stage (diagnosis/sample)	Previous treatment	Response
CLL1	0/III	R-FC	Complete remission
CLL2	0/IV	F; C; COP; R-FC	No response
CLL3	IV/0	R-FC	Complete remission
CLL4	I/III	Chlorambucil	Partial remission
CLL5	0/0	Chlorambucil	Partial remission
CLL6	0/0	None	
CLL7	0/IV	R-COP; R-chlorambucil; R-BENDA/Idelalisib	No response
CLL8	IV/IV	Chlorambucil	No response
CLL9	0/0	Splenectomy	Partial remission

*R, rituximab; F, fludarabine; C, cyclophosphamide; O, vincristine; P, prednisone; BENDA, bendamustine*.

Next, we analyzed whether HLA-I expression in the LCLs used to activate NK cells might influence their anti-leukemic potential. To this aim we compared the cytotoxic activity of NK cells from 4 independent donors, activated either with HLA-I^+^ R69 LCLs or with HLA-I^−^ 721.221 cells, against B-CLL cells from four different patients. As shown in Figure 1, although with some individual variations, the cytotoxic potential of activated NK cell did not depend on HLA-I expression in stimulating LCLs (Figure 1B). Thus, we employed NK cells isolated from PBMC cultures activated for 5 days with R69 LCLs in the next experiments.

### Expression of NK Cell Ligands by B-CLL Cells

Our data confirm that LCL-activated NK cells are able to kill B-CLL cells. However, we have previously found that the cytotoxic potential of allogeneic NK cells against B-CLL cells varies depending on the NK cell donor/B-CLL patient pair (51).

**Figure 2:**
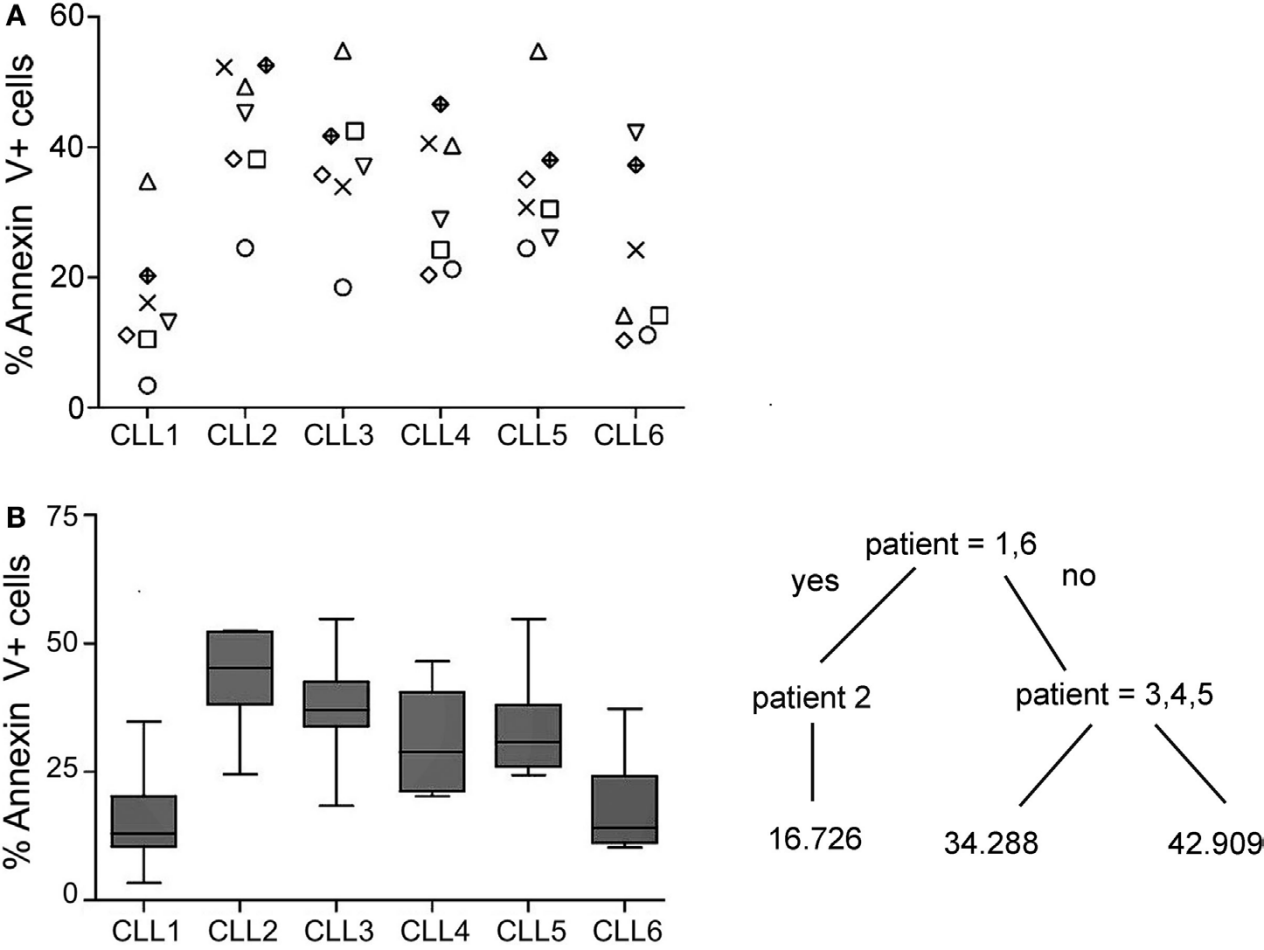
**Analyses of the cytotoxic potential of activated allogeneic NK cells employing six CLL patients and seven NK cell donors**. Cells from B-CLL patients were incubated with R69-activated NK cells (MACS enriched, >90% CD56^+^CD3^−^ cells; CTGreen labeled) from seven independent donors for 4 h at 9:1 effector:target ratio in two independent experiments as described in Section “Materials and Methods.” Subsequently, PS traslocation (annexin V DY634) and membrane permeabilization (7AAD uptake) were analyzed by flow cytometry in the cell population negative for CTGreen. **(A)** The graph represents the % of annexin V positive cells in the 42 combinations (six B-CLL × seven NK). Annexin V^+^ cells represent the % of Annexin V^+^7AAD^−^ plus AnnexinV^+^7AAD^+^ cells. Each symbol represents a NK cell donor. All NK cell donors were incubated with every B-CLL sample. **(B)** Cell death (Annexin V^+^) in every B-CLL sample was represented in a boxplot where median ± SD is indicated. Statistical analysis was performed employing a regression tree in which patient samples are clustered according to their similarity of sensitivity to NK cell cytotoxicity.

Thus, experiments were set up to address the basis for the variable susceptibility of B-CLL cells to activated NK cells. Seven NK cell donors and six B-CLL patients were selected, and the level of cell death was evaluated in all combinations (42). Four and three different NK cells donors were blindly incubated with the six B-CLL patients on two different days. As shown in Figure 2A, the level of cell death varied depending on the pair of NK cell donors and B-CLL patients incubated in agreement with our previous results (51). Some donors like the one represented with circles presented a similar profile of killing against all CLL samples. However, this was not a general trend since others like the ones represented with triangles, diamonds, or inverted triangles presented a high variability depending on the CLL sample. Indeed, the profile of killing between the different NK cell donors was statistically similar (Supplementary figure). These killing profiles were not dependent on the level of NK cell activation since all of them similarly killed Jurkat cells and expressed comparable levels of granzyme B, NKG2D, and NCRs (NKp30, Nkp44, and Nkp46) (data not shown). Thus, *a priori*, this variability could be partly explained by (i) the degree of HLA–KIR mismatch between donor and patient as found in AML and ALL or (ii) the intrinsic resistance of cells from some B-CLL patients to activated NK cells.

**Figure 3:**
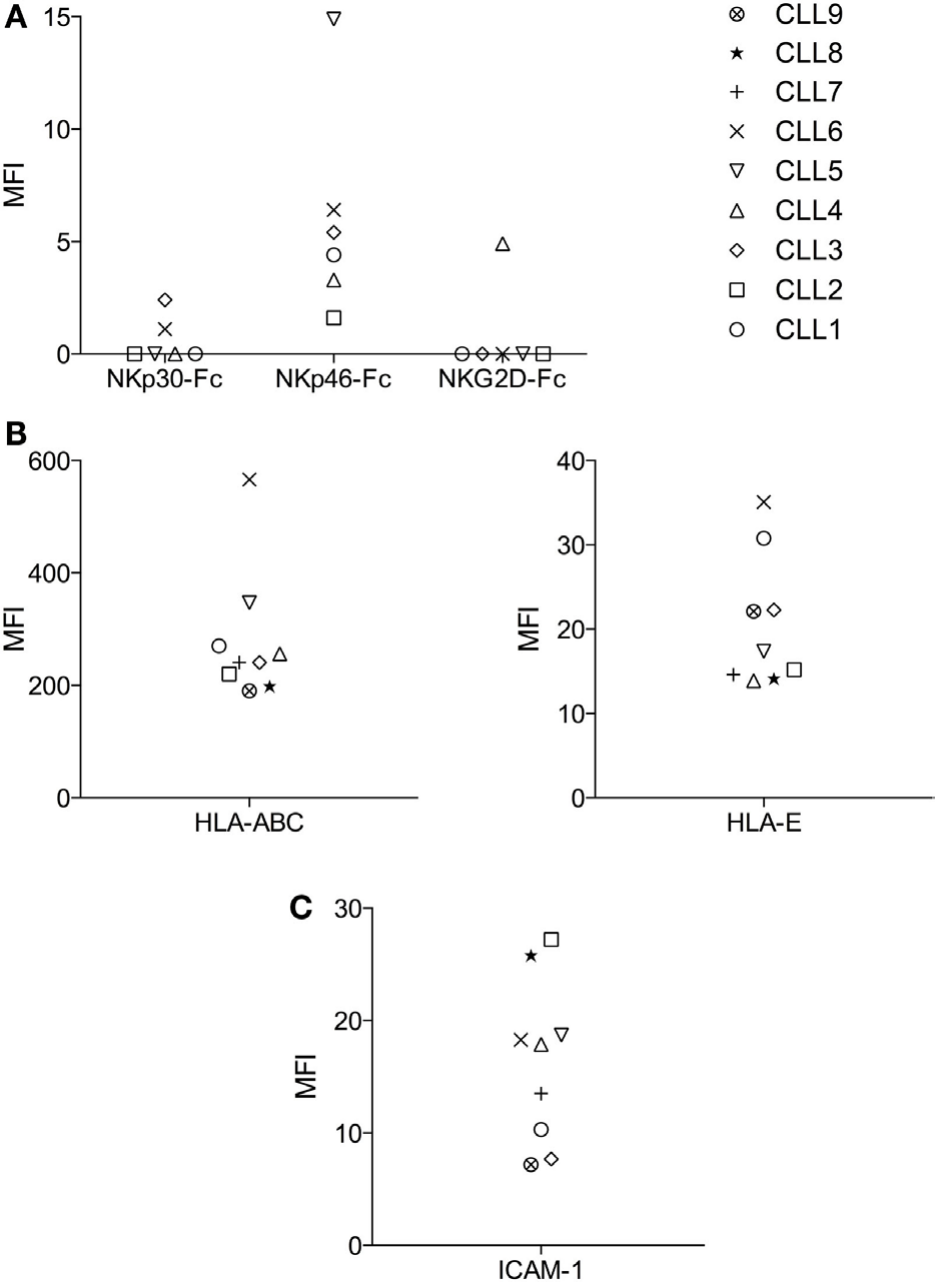
**Analyses of the expression of activating and inhibitory ligands of NK cell receptors in B-CLL cells**. Expression of NKp30, NKp46, and NKG2D activating ligands using Fc quimeras **(A)**, HLA-ABC and HLA-E inhibitory ligands **(B)**, and the adhesion molecule ICAM-1 **(C)** using specific antibodies were analyzed in B-CLL cells by flow cytometry as described in Section “Materials and Methods.” The Mean fluorescence intensity (MFI) of every B-CLL sample is represented in the graphs. MFI = (MFI specific Ab) − (MFI isotype control).

As shown in the regression-tree analysis in Figure 2B, some B-CLL cells like CLL1 and CLL6 are more resistant than others to activated NK cells, irrespectively of the NK cell donor, supporting our second hypothesis. Thus, we decided to analyze the reason for the increased resistance of CLL1 and CLL6 to activated NK cells. First, we analyzed whether this could be related to the level of expression of ligands recognized by the NK cell receptors NKG2D, NKp30, NKp46, KIRs, and LFA-1. As shown in Figure 3A, the expression of NKG2D and NKp30 ligands were very low in most CLL samples, and only some CLLs expressed higher levels of NKp46 ligands. Jurkat cells expressed ligands for all these receptors, validating our approach. Next, we analyzed the expression of the adhesion molecule ICAM-1 and of HLA-I using both an antibody (clone W6/32) that recognizes all HLA-I molecules and another one specific for HLA-E. As shown in Figure 3B, all cells expressed HLA-I (ABC/E/F/G and E) and ICAM1, although the level of expression was quite variable. Of note, the level of expression of HLA-ABC and HLA-E in CLL1 and CLL6 was higher in comparison with the other B-CLL samples, suggesting that this expression could be related to the increased resistance of these samples. However, blocking of CD94 in NK cells, a protein required for the inhibitory signaling of the HLA-E ligand NKG2A, by using specific antibodies did not significantly enhance NK cell-mediated cytotoxicity (data not shown) suggesting that HLA-E expression in CLL1 and CLL6 was not the main responsible for NK cell resistance.

The adhesion molecule ICAM-1 was expressed by all B-CLL samples (Figure 3C), and the level of expression did not correlate with NK cell susceptibility. Although we did not test other adhesion molecules involved in NK cell recognition like LFA-3, we have previously shown that blocking ICAM-1/LFA-1 interaction almost completely eliminate NK cell cytotoxicity in hematological cancer cells (including B-CLL) [(55) and data not shown] suggesting a minor role for other adhesion molecules.

### KIR–HLA Receptor–Ligand Mismatch

As mentioned above KIR–HLA mismatch has been shown to promote NK cell-mediated elimination of some hematological neoplasias, including acute monocytic and lymphocytic leukemias, and to a lesser extent multiple myeloma (56). However, this effect is not clear for B-CLL, and it could help to explain the different susceptibility of some B-CLL cells. Thus, we analyzed whether mismatches between NK cell donor KIRs and patient HLA ligands (HLA-A3/A11, -Bw4, -C1/2) would explain the differences observed. To this end, we determined the HLA epitopes for the seven NK cell donors and the six B-CLL patients. The results of these analyses are shown in Table 2 where the expression of known ligands for KIR3DL2 (A3/A11), KIR3DL1 (Bw4), KIR2DL1 (C2), and KIR2DL2/2DL3 (C1) are indicated.

**Table 2:** **KIR ligands expressed in CLL patients and NK cell donors used in the study of compatibility**.

DNA	lig 3DL2	lig 3DL1	lig 2DL1	lig 2DL2 2DL3
CLL1	−	+	+	+
CLL2	−	−	+	+
CLL3	−	+	+	−
CLL4	−	+	−	+
CLL5	−	−	+	+
CLL6	+	+	−	+
CLL7	−	+	+	+
NK1	−	+	+	−
NK2	−	+	+	+
NK3	+	+	−	+
NK4	−	+	+	+
NK5	−	+	−	+
NK6	−	+	+	−
NK7	−	+	+	+

**Figure 4:**
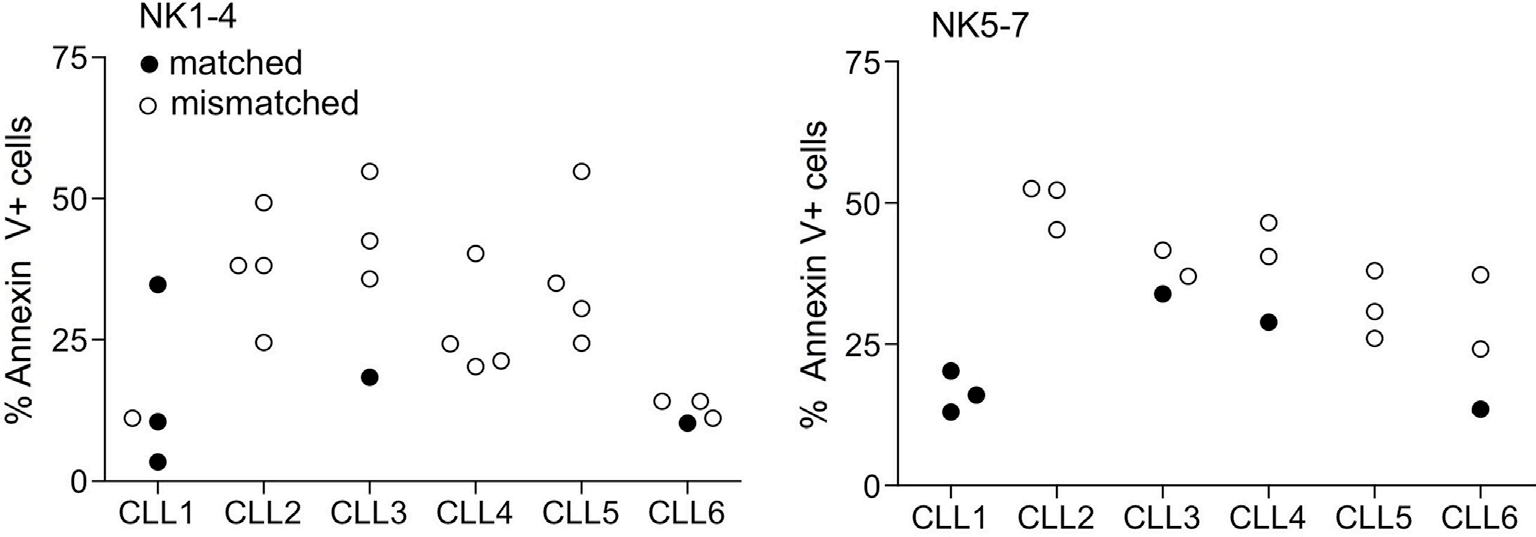
**Correlation between matched B-CLL/NK cell combinations and cell death**. The data in Figure 2 were now represented as matched and mismatched combinations. The graph represents the % of annexin V^+^ cells in the 42 combinations (six B-CLL × seven NK) as described in legend to Figure 2 separated in two independent experiments (NK1-4 and NK5-7). White and solid symbols correspond to mismatched and matched combinations, respectively. Statistical analyses and results are described in Tables 4 and 5.

Based on the degree of KIR ligand compatibility in donor to patient direction, we predicted matched (compatible) and mismatched (incompatible) combinations in the 42 NK-CLL pairs and analyzed whether the degree of mismatch significantly correlated with the susceptibility observed in the B-CLL samples. A summary of the percentage of matched (0) and mismatched (1) combinations is shown in Table 3. As expected, since the experimental design followed a double blind protocol, in which HLA genotype and mismatch was analyzed after testing NK cell-mediated cytotoxicity, a higher number of unmatched than matched combinations were analyzed. However, as shown in Figure 3, matched and unmatched NK-CLL combinations were tested at the same time, which partially compensate this potential limitation. As shown in Figure 4, except for one NK cell donor in CLL1, matched NK cell donors induced the lowest level of cell death in every B-CLL case, although in some instances differences were low. The median cell death in compatible B-CLL/NK cell combinations was 18%, and this value increased to 34% in mismatched combinations (Table 4). These differences became statistically significant using a one mixed-model analysis of variance (ANOVA). The model controlled for the within-subject nature of the seven NK cell donors by including random effects for B-CLL patient and B-CLL patient × NK cell interaction. Thus, although the statistical significance was close to 0.05, it should be noted that the model used for these analyses is very conservative taking into account that there is a non-controlled intrinsic variability in the components within each group (NK cell donors and B-CLL patients). Thus there was a good correlation between mismatching and NK cell cytotoxicity supporting that NK cell alloreactivity explains the variability observed in each B-CLL sample.

**Table 3:** **Distribution of matched (0)/mismatched (1) combinations for each CLL patient**.

CLL	Compatibility	
	0 (%)	1 (%)
1	6 (86)	1 (14)
2	0 (0)	7 (100)
3	2 (29)	5 (71)
4	1 (14)	6 (86)
5	0 (0)	7 (100)
6	2 (29)	5 (71)

**Table 4:** **Statistical analyses of the difference in cell death between matched (0) and mismatched (1) groups**.

	Compatibility			
	0 (%)		1 (%)	
Min	3.4		11.2	
1st qu	11.8		24.83	
Median	16		37	
Mean	18.49		34	
3rd qu	24.65		34	
Max	34.80		54.75	

	**numDF**	**denDF**	***F*-value**	***p*-value**

Intercept	1	35	74	0
Incompatible	1	35	4.262	0.046

### Poor Prognosis B-CLL Cells Are More Susceptible to Activated NK Cells than Good Prognosis B-CLL Cells

Although mismatch partly explained the variability observed in the different B-CLL/NK cell combinations, our results also suggest that in each B-CLL case there is/are intrinsic characteristic/s that regulate/s the susceptibility of B-CLL cells to NK cells which is independent on the expression of NK cell receptor ligands.

When we analyzed retrospectively the clinical evolution (response to treatment) of these patients, we realized that except for CLL3, paradoxically, samples from patients with a better response (CLL1) or in which no treatment was required (CLL6) were those more resistant to NK cell cytotoxicity (Table 1; Figure 2B). In contrast, the patient that did not respond to treatments (CLL2) was the most susceptible to NK cells. Based on this observation, we established a new experiment to analyze in more detail whether the prognosis of CLL patients correlated with the susceptibility of B-CLL cells to NK cells. Prognosis was established by determination of *TP53* and *IGHV* mutational status as previously shown (16–19). Three different groups of B-CLL patients were established retrospectively according to the expression of wild-type (wt) or mutated/deleted (mut) *TP53* and wt or mut *IGHV*: *TP53*^wt^*IGHV*^mut^ (nine patients), *TP53*^wt^*IGHV*^wt^ (eight patients), and *TP53*^mut^*IGHV*^wt^ (five patients) as good, intermediate, and poor prognostic groups (Table 5). The samples of these groups were blindly incubated with NK cells from several donors randomly selected and the level of cell death was determined.

**Table 5:** **Characteristics of CLL patients used in the study of susceptibility in relation to prognosis**.

Sample	Stage (diagnosis/sample)	Previous treatment	Response	FISHCYT^a^	IGHV status	TP53/IGHV status
LC15001	I/I	N		13q	IGHV4-34	P53wt/IGHVm
LC15004	I/I	N		13q	IGHV3-74	P53wt/IGHVm
LC15006	I/I	N		N	IGHV1-2	P53wt/IGHVm
LC15007	0/0	N		N	IGHV4-39	P53wt/IGHVm
LC15012	I/I	N		N	IGHV3-49	P53wt/IGHVm
LC15014	I/IV	BENDA	Partial remission (dead)	13q	IGHV4-34	P53wt/IGHVm
LC15015	0/0	N		N	IGHV3-30	P53wt/IGHVm
LC15030	I/II	Chl	Partial remission (dead)	11q	IGHV3-74	P53wt/IGHVm
12909A	I/I	N		N		P53wt/IGHVm
LC15003	I/II	N		12	IGHV4-39	P53wt/IGVHwt
LC15008	II/II	R-Chl	Partial remission (dead)	13q	IGHV4-39	P53wt/IGHVwt
LC15009	0/0	N		N	IGHV3-20	P53wt/IGHVwt
LC15010	II/II	Chl	No response	12	IGHV1-18	P53wt/IGHVwt
LC15011	I/I	R-FC LITE	No response (dead)	12	IGHV4-39	P53wt/IGHVwt
LC15013	II/II	R-FC	Complete remission	11q	IGHV4-b	P53wt/IGHVwt
LC15032	II/II	R-FC	Partial remission	N	IGHV3-21	P53wt/IGHVwt
LC15061	0/0	GA101- Chl	Partial remission nodular	13q	IGHV1-2	P53wt/IGHVwt
LC15033	0/I	R-BENDA	Complete remission	p53	IGHV1-69	P53m/IGHVwt
LC15055	II/II	BENDA	Partial remission	p53	IGHV1-69	P53m/IGHVwt
16238A	0/IV	R-FCM + Rm	Complete remission	p53	IGHV3-9	P53m/IGHVwt
17114A	II/III	R-FC	No response (dead)	p53	IGHV4-39	P53m/IGHVwt
15267A	0/IV	R-FC	No response (dead)	p53	IGHV3-7	P53m/IGHVwt

*R, rituximab; F, fludarabine; C, cyclophosphamide; BENDA, bendamustine; Chl, chlorambucil; M, mitoxantrone; GA101, Obinutuzumab; LITE, increased R dose and reduced FC dose; N, none*.

*^a^Genetic abnormalities found by FISH: trisomy 12, 11q and 13q deletion: p53 deletion/mutation*.

**Figure 5:**
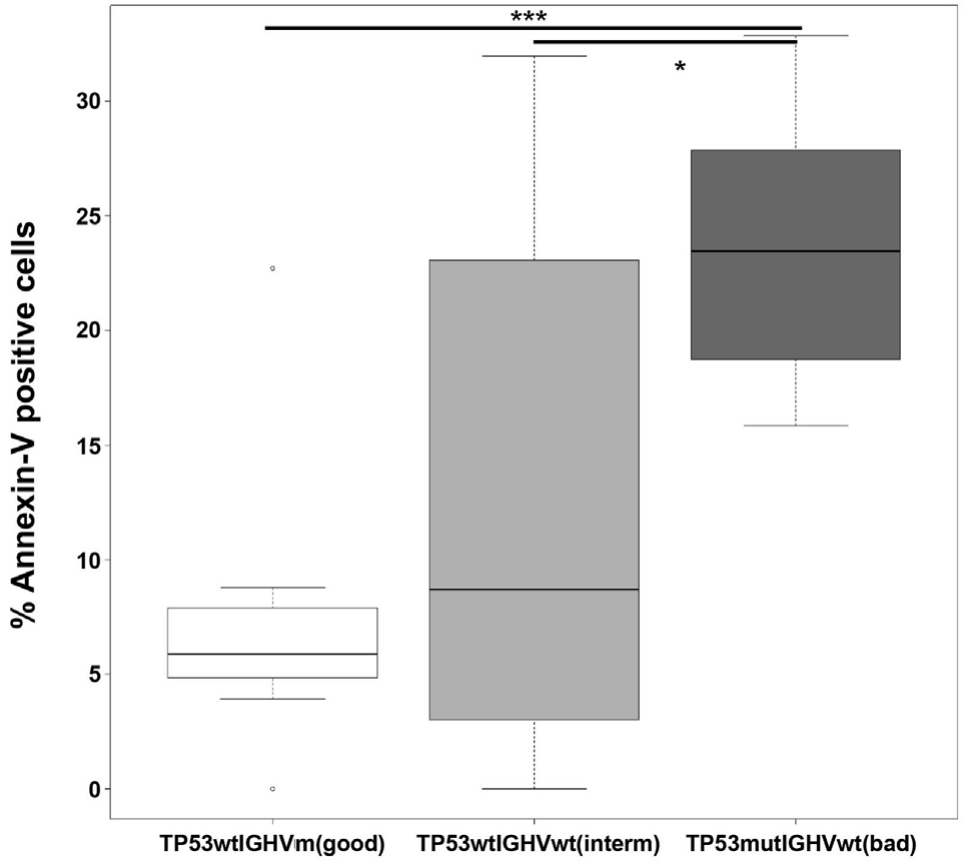
**Poor prognosis B-CLL cells are more susceptible to allogeneic R69-LCL-activated NK cells than good prognosis samples**. R69-LCL-activated allogeneic NK cells (MACS enriched, >90% CD56^+^CD3^−^ cells; CTGreen labeled) were incubated for 4 h at 9:1 effector:target ratio with B-CLL cells from three groups of patients classified according to prognosis based on the mutational status of *TP53* and *IGHV* (good: *TP53*^wt^*IGHV*^mut^, *n* = 9; intermediate: *TP53*^wt^*IGHV*^wt^, *n* = 8; bad: *TP53*^mut^*IGHV*^wt^, *n* = 5). Subsequently, PS traslocation (annexin V DY634) and membrane permeabilization (7-AAD uptake) were analyzed by flow cytometry in the cell population negative for CTGreen. Annexin V^+^ cells represent the % of AnnexinV^+^7AAD^−^ plus AnnexinV^+^7AAD^+^ cells. Every B-CLL sample was incubated with NK cells from the several healthy donors and the % of Annexin V^+^ cells for every B-CLL sample were represented in a boxplot where median ± SD is indicated. Statistical analysis was performed by comparing the means of every group using 1-way ANOVA with Tukey HSD *post hoc* test; ns, not significant, *p < 0.05, ***p < 0.001.

As shown in Figure 5, the susceptibility of B-CLL cells to NK cells was clearly enhanced in the worse prognostic group in comparison with good and intermediate prognosis. Expression of wt *IGHV* in the context of wt *TP53* also increased the susceptibility of B-CLL cells to NK cells although did not reach statistic significance.

## Discussion

Allogeneic NK cells have been shown to be effective in the treatment of poor prognosis acute myeloid and lymphoid leukemia (AML and ALL) (34, 37–42). Here, we show that allogeneic activated NK cells also recognize B-CLL cells and that, as shown for AML and ALL, our results suggest that recognition might be partially dependent on HLA mismatch. In addition, we show that allogeneic NK cells are able to efficiently kill leukemic cells from B-CLL patients with very poor prognosis categorized according to expression of mutated *TP53* and wt *IGHV*. Notably, a worse prognosis was associated with an increased susceptibility of B-CLL cells to activated NK cells suggesting that those tumors expressing more aggressive phenotypes due to mutations may be good targets for NK cell immunotherapy. Indeed, it has recently been shown that B-CLL cells expressing wt *IGHV* present a higher number and a different pattern of somatic mutations, conferring those associated with *IGHV* a more aggressive and drug-resistant phenotype (15, 57, 58). Although we do not have yet a molecular explanation for this finding, it suggests that activated allogeneic NK cells could be a good alternative to treat them. Indeed, these results are somehow in agreement with previous findings indicating that cells with a higher resistance like undifferentiated/stem like tumors are more susceptible to NK cells (59–63). Since two different cohorts of patients were used for these studies, the formal proof that a combination of mismatch and prognosis predicts the sensitivity of B-CLL cells to allogeneic NK cells is still lacking. However, our data suggest that both factors should be taken into account when selecting therapies against bad prognosis B-CLL.

Controversial findings have been reported on the susceptibility of B-CLL cells to NK cells. Some studies found that activation of NK cells with IL2 increased its cytotoxic potential against B-CLL cells (43–45), by contrast others reported opposite results (46–48). However, these studies did not analyze the degree of HLA compatibility between donor NK cells and B-CLL cells, which could explain these *a priori* contradictory results. A general explanation for the relative resistance of B-CLL cells to syngeneic NK cells is the low expression of NKR ligands in B-CLL cells together with a high expression of classical HLA-I genes (64–66). Although we have not specifically identified which NKRs are involved in the elimination of B-CLL cells, our data suggest that allogeneic activated NK cells recognize and kill B-CLL cells even expressing low levels of activating NKR ligands. An enhanced NKR expression after activation as previously found (51) and a reduction of inhibitory signals due to HLA mismatch would promote the allogeneic NK cell response against B-CLL cells. This strategy is being currently used to develop therapeutic alternatives like blocking KIR with the antibody, Lirilumab (67). In this case the effect maybe more limited due the inability of patient NK cells to be reactivated against leukemic cells. Our results suggest that combining activated NK cells with blocking KIR antibodies might enhance the therapeutic potential of these therapies.

Another critical factor that may explain the differences regarding the susceptibility of B-CLL cells to NK cells is the use of NK cells activated under different protocols. We have previously found the stimulus employed to activate NK cells is critical in predicting the effectiveness of NK cells against resistant hematological cancer cells (51, 52). Thus, in contrast to studies mainly employing cytokines (IL-2 and/or IL-15) to activate NK cells, we have used here an EBV-transformed B-LCL as feeder cells. This protocol has been shown to enhance NK-mediated cytotoxicity against hematological neoplasia more efficiently than cytokines even in combination with K562 feeder cells (51, 52).

Following this protocol, we have analyzed the molecular determinants that might explain the relative resistance of B-CLL to NK cells. First, we have confirmed that activation is a prerequisite to generate allogeneic NK cells able to kill B-CLL cells. We ruled out that the activation level of NK cells from the different donors was responsible for the different susceptibility of B-CLL [Data not shown and (51)]. In addition, we did not find a clear correlation between expression of ligands for the major activating NK cell receptors (e.g., NKG2D, NKp30, and NKp46) or the ICAM-1 adhesion molecule and B-CLL susceptibility to NK cells. Concerning the major ligands for NK inhibitory receptors, a good correlation between HLA-ABC and HLA-E expression and B-CLL susceptibility to NK cells was observed. However, a more rigorous analysis of HLA-ABC expression should be performed in a higher number of samples to reach a clear conclusion.

Employing a total of 42 NK donor-B-CLL patient combinations, we obtained data suggesting that HLA mismatching was associated with the level of susceptibility of every B-CLL case. However to definitively confirm it, the expression of KIRs at the protein level should be also analyzed in a higher number of combinations, as a receptor–ligand model could improve the prediction of NK cell effectivity (68). Despite these potential limitations, our results underline the importance of selecting a suitable NK cell donor to treat a given B-CLL patient. In particular the analysis of HLA mismatch would be useful to readily exclude NK cell donors with low effectivity (compatible). Yet, testing susceptibility to NK cells from mismatched donors *in vitro* is mandatory to select a good NK cell donor, since B-CLL from some patients are inherently more resistant than others, irrespectively of HLA mismatch.

The general resistance of some B-CLL patients could be due to previously reported immune evasion mechanisms, i.e., shedding of NK cell receptor-soluble ligands (66). Although these authors did not test the role of HLA mismatch in B-CLL resistance, and we have not formally tested this potential evasion mechanism, our data comparing the susceptibility of B-CLL cells from patients with good, intermediate, and bad prognosis suggest that the low susceptibility of some B-CLL patients is paradoxically related with a less aggressive cancer cell phenotype.

Irrespective of which signals regulate NK cell recognition of B-CLL cells, it seems clear that expression of poor prognosis markers like mutated or deleted *TP53* and wt *IGHV*, related to low life expectancy, may be converted into a chance to employ activated allogeneic NK cells to treat aggressive B-CLL phenotypes. Studies employing larger cohort or patients and clinical trials will be required to confirm the therapeutic value of our findings. Encouraging the development of these trials our conclusions are supported by recent findings indicating that allogeneic stem cell transplantation is a good option for poor prognosis B-CLL if the balance between GVL effect and GVHD is evaluated properly (69).

## Author Contributions

DS-M performed most experimental work, analyzed results, and wrote the first draft of the manuscript. PL performed experimental work and analyzed results. NG, GA, LP, and JM selected and provided LLC samples, analyzed clinical data, and wrote the manuscript. AM provided NKR-chimeras. EC, MM, and CV performed immunophenotyping of NK cell donors and patient samples and the mismatch study. AA, LM-L, and MV discussed the experimental data and wrote the manuscript. JP designed, supervised, and evaluated the experiments and wrote the final version of the manuscript. All authors read and approved the manuscript.

## Conflict of Interest Statement

The authors declare that the research was conducted in the absence of any commercial or financial relationships that could be construed as a potential conflict of interest.
