# Supplementary figures and images for: Activated Allogeneic NK Cells Preferentially Kill Poor Prognosis B-Cell Chronic Lymphocytic Leukemia Cells
Source: Front Immunol. 2016 Oct 27;7:454. doi: 10.3389/fimmu.2016.00454 (PMC5081347; doi:10.3389/fimmu.2016.00454)

**Comparison of the cytotoxic activity of the different NK cell donors against B-CLL samples (6).**

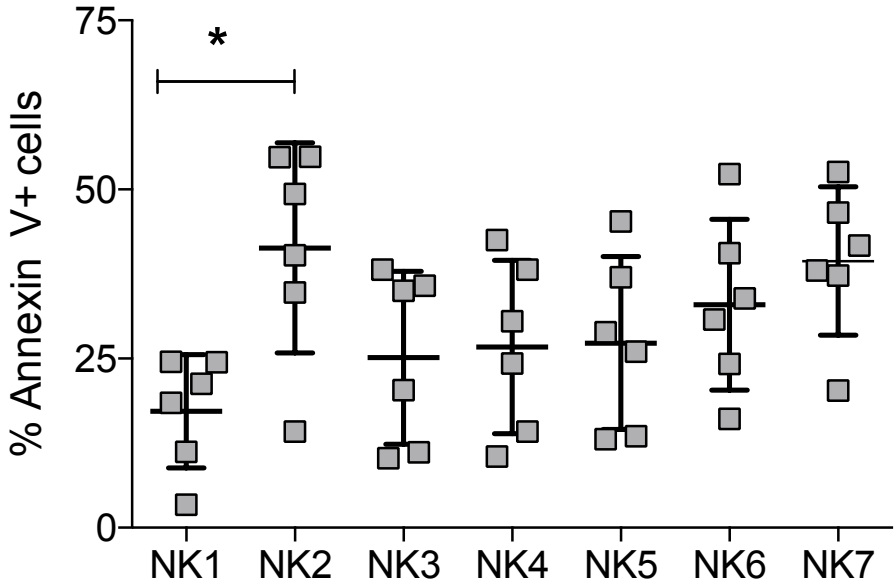

Supplement: Supplementary file 1 [file Image_1.PDF]
